# Which patients do not return to work after total knee arthroplasty?

**DOI:** 10.1007/s00296-016-3512-5

**Published:** 2016-06-24

**Authors:** P. Paul F. M. Kuijer, Arthur J. Kievit, Thijs M. J. Pahlplatz, Truus Hooiveld, Marco J. M. Hoozemans, Leendert Blankevoort, Matthias U. Schafroth, Rutger C. I. van Geenen, Monique H. W. Frings-Dresen

**Affiliations:** 1 People and Work Outpatient Clinic, Coronel Institute of Occupational Health, Academic Medical Center, University of Amsterdam, PO Box 22660, 1100 DD Amsterdam, The Netherlands; 2 Orthopaedic Research Center Amsterdam, Academic Medical Center, University of Amsterdam, Amsterdam, The Netherlands; 3 MOVE Research Institute, Department of Human Movement Sciences, Vrije Universiteit Amsterdam, Amsterdam, The Netherlands; 4 Department of Orthopaedics, Amphia Hospital, Breda, The Netherlands

**Keywords:** Arthroplasty, Replacement, Employment, Physical activity, Body mass index, Pain, Prognosis

## Abstract

Total knee arthroplasty (TKA) is increasingly being performed among working patients suffering from knee osteoarthritis. Two out of ten patients do not return to work (RTW) after TKA. Little evidence is available about these patients to guide clinicians. Therefore, this study investigates patients’ characteristics associated with no RTW. A multicenter retrospective cohort study was performed among working patients having undergone a primary TKA during 2005–2010. The following preoperative characteristics were assessed: age at surgery, sex, comorbidity, body mass index (BMI), preoperative sick-leave duration, patient-reported work-relatedness of knee symptoms, and physical job demands. In addition, the Knee injury and Osteoarthritis Outcome Scores (KOOS) after TKA were assessed. Backward stepwise logistic regression analyses were performed to predict no RTW. Seven hundred and sixty**-**four patients were approached, and 558 patients (73 %) responded. One hundred and sixty-seven met the inclusion criteria and 46 did not RTW. A preoperative sick-leave duration >2 weeks (OR 12.5, 90 % CI 5.0–31.5) was most strongly associated with no RTW. Other associations found were: female sex (OR 3.2, 90 % CI 1.3–8.2), BMI ≥ 30 (OR 2.8, 90 % CI 1.1–7.1), patient-reported work-relatedness of knee symptoms (OR 5.3, 90 % CI 2.0–14.1), and a physically knee-demanding job (OR 3.3, 90 % CI 1.2–8.9). Age and KOOS were not associated with no RTW. Especially obese female workers, with a preoperative sick-leave duration >2 weeks, who perform knee-demanding work and indicate that their knee symptoms are work-related have a high chance for no RTW after TKA. These results stress the importance of a more timely referral for work-directed care of patients at risk for no RTW after TKA.

## Introduction

Total knee arthroplasty (TKA) is highly effective in treating pain and loss of function caused by knee osteoarthritis (OA) [1]. Historically, TKA has mostly been performed in older retired patients. However, recent studies show that more patients are of working age. In the USA, more than 50 % of the primary TKA patients are younger than 65 years old by 2016 [2]. In the Netherlands in 2013, the incidence of TKA surgery among 45- to 64-year-old patients more than tripled between 1995 and 2005 [3]. The prediction for the Netherlands is that the number of patients receiving TKA will increase by about 300 % to 57,900 annually in 2030 [3]. For the UK, these figures increase to at least 118,666 in 2035, with 11 % being younger than 60 years old [4]. At the same time, the age for retirement is expected to rise in the Netherlands and other western countries. Therefore, an increasing number of knee OA patients will need to be able to RTW after TKA surgery. However, based on data from 11 studies, 307 out of 1417 patients working before TKA surgery (22 %) did not RTW [5, 6]. Given the increasing numbers of TKAs performed among working knee OA patients worldwide, this ratio reflects a large group of workers.

RTW is a multidimensional concept with disease- and non-disease-specific prognostic factors [7]. Remarkably little disease-specific evidence is available for clinicians to guide RTW. Only two studies, including a total of 332 TKA patients, have assessed TKA-specific factors associated with RTW based on multivariate analyses [8, 9]. Two factors hindered RTW in both studies: sex and physical job demands. Both studies agreed on high physical job demands. For sex, one study found that being a male hindered RTW [9], while the other study found the same for being a female [8]. To ensure an appropriate and timely diagnosis for additional work-directed care, knowledge on characteristics of TKA patients at risk for no RTW is urgently needed [10–13]. Therefore, this study investigates patient characteristics associated with no RTW after TKA.

## Methods

### Study design and setting

A multicenter retrospective cohort study was performed in 2012 [14]. Patients eligible for inclusion had undergone a primary Vanguard TKA between 2005 and 2010 and were working preoperatively within 2 years, and had a follow-up of at least 2 years. The study received approval from the Medical Ethics Committee of the Academic Medical Center in Amsterdam (AMC), the Netherlands. A total of 764 eligible patients who had undergone TKA received an invitation from two Dutch hospitals to participate. Patient informed consent was obtained.

### Data collection

Patient characteristics and RTW were obtained from the patient file and a questionnaire sent in 2012.

### Patient characteristics associated with no RTW

The included patient characteristics for RTW were based on studies on work participation among patients with TKA and hip arthroplasty [5, 6, 8, 9, 15, 16]. From the patients’ files we retrieved: sex (female or male), age at surgery (years), body mass index (BMI, kg/m^2^), classified in two categories <30 “normal” and ≥30 “obese,” and comorbidity according to the first three categories of the American Society of Anesthesiologists physical status classification before surgery: healthy, mild systemic disease, and severe systemic disease. In the questionnaire, we assessed the Knee injury and Osteoarthritis Outcome Score (KOOS) after TKA for pain, other symptoms, activities of daily living (ADL), functioning in sports and recreation (Sport/Rec), and knee-related quality of life (QoL). All subscales received a sum score between 0 and 100, with 0 representing extreme knee problems and 100 representing no knee problems. The values were dichotomized based on care-seeking behavior: pain ≤86.1; symptoms ≤85.7; ADL ≤ 86.8; Sport/Rec ≤85.0; and QoL ≤ 87.5 [17]. In addition, the following characteristics were retrospectively assessed in the questionnaire: preoperative sick leave before TKA surgery (0–2, 2–4, 1–3 months, 3–6 months, or more than 6 months, dichotomized into 0–2 and >2 weeks), and whether the patient thought their work had caused or aggravated their knee symptoms, finally resulting in TKA (patient-reported work-relatedness of knee symptoms dichotomized into yes for “totally agree”, “agree” and “neither disagree nor agree”, and no for “disagree” and “totally disagree”). The kind of job patients performed before TKA surgery was classified based on job title by two occupational health experts into light-, medium-, and heavy knee-demanding work based on work-related risk factors for knee OA [5, 18].

### No return to work

Patients responding affirmatively to the question “I didn’t get back to work” after TKA surgery in the follow-up questionnaire were classified as not having returned to work (no RTW). The reference category consisted of patients that did return to work.

### Data analyses

Backward stepwise regression analysis was performed to build a model for no RTW using IBM SPSS Statistics 22. Due to the relatively small number of patients included (*n* = 332) in the only two other prognostic multivariate studies on RTW specific for TKA, all factors were a priori included in the regression analysis after controlling for multicollinearity (variance inflation factors >10 and tolerance <0.1). To overcome bias due to differences in follow-up time between TKA surgery and filling in the questionnaire, this period was also included in the regression analysis. In the case of factors with more than two categories, dummies were used. odds ratios (OR) were calculated, including 90 % confidence intervals (CI) to prevent possibly relevant clinical variables from being opted out.

## Results

### Patient characteristics before surgery

Five hundred and fifty-eight patients of the contacted 764 patients responded (response rate 73 %), of which 78 patients did not wish to participate. The remaining 480 participants all filled in the questionnaire. The mean follow-up time of the questionnaire survey was 3.8 years (SD 1.3) after TKA surgery. Of these 480 patients, 167 worked before TKA surgery and were included in the analysis (Table 1). The male/female ratio was 49 %:51 %. The mean age at TKA surgery was 60 years (SD 8), and the mean age at follow-up was 64 years (SD 8). Fifty-eight percent had a BMI < 30 kg/m^2^ and 42 % a BMI ≥ 30. Sixty-five percent of the patients had a preoperative sick leave of 2 weeks or less. Forty-eight percent performed light-, 32 % medium-, and 20 % heavy knee-demanding work before TKA surgery. Thirty-one percent of the TKA patients were of the opinion that their work had caused or aggravated their knee symptoms. Out of the five postoperative KOOS’s, pain had the most favorable outcome: a mean of 79.6 for the group as a whole (Table 1).

**Table 1:** Pre- and postoperative characteristics [mean, standard deviation (SD) or number (*n*) and percentage (%)] of TKA patients of the group as a whole (All) and specified for the patients that returned (RTW) and did not return to work (no RTW)

	All *n* = 167	RTW *n* = 121	No RTW *n* = 46
*Preoperative*			
Sex (*n*, %)			
Male	82 (49.1)	63 (52.1)	19 (41.3)
Female	85 (50.9)	58 (47.9)	27 (58.7)
Age at surgery (mean, SD)			
Year	59.7 (8.4)	58.8 (8.3)	62.1 (8.3)
<60 (*n*, %)	85 (50.9)	69 (57)	16 (35)
>=60 (*n*, %)	82 (49.1)	52 (43)	30 (65)
Body height (mean, SD)			
m	1.74 (0.09)	1.75 (0.09)	1.72 (0.11)
Body weight (mean, SD)			
kg	88.7 (15.6)	89.2 (15.6)	87.5 (15.6)
Body mass index (*n*, %)			
<30 kg/m^2^	96 (58.5)	70 (59.8)	25 (54.3)
≥30 kg/m^2^	68 (41.5)	47 (40.2)	21 (45.7)
ASA classification (*n*, %)			
Type 1	51 (31.1)	38 (31.9)	13 (28.9)
Type 2	93 (56.7)	68 (57.1)	25 (55.6)
Type 3	20 (12.2)	13 (10.9)	7 (15.6)
Preoperative sick leave (*n*, %)			
0–2 weeks	108 (65.5)	95 (79.2)	13 (28.9)
2–4 weeks	6 (3.6)	4 (3.3)	2 (4.4)
1–3 months	14 (8.5)	10 (8.3)	4 (8.9)
3–6 months	11 (6.7)	5 (4.2)	6 (13.3)
>6 months	26 (15.8)	6 (5.0)	20 (44.4)
Knee-demanding work (*n*, %)			
Light	66 (48.2)	53 (53.0)	13 (35.1)
Medium	44 (32.1)	25 (25.0)	19 (51.4)
Heavy	27 (19.7)	22 (22.0)	5 (13.5)
Work-relatedness knee complaints (*n*, %)			
No	115 (68.9)	92 (76.0)	23 (50.0)
Yes	52 (31.1)	29 (24.0)	23 (50.0)
*Postoperative*			
KOOS Pain (mean, SD)			
0–100	79.6 (22.3)	81.9 (20.5)	73.6 (25.8)
≤86.1 (*n*, %)	69 (41.6)	49 (40.5)	20 (43.5)
KOOS Symptoms (mean, SD)			
0–100	72.6 (19.8)	73.8 (19.5)	69.4 (20.6)
≤85.7 (*n*, %)	110 (66.3)	77 (63.6)	33 (71.7)
KOOS ADL (mean, SD)			
0–100	77.5 (22.7)	80.6 (19.8)	69.4 (27.7)
≤86.8 (*n*, %)	166 (53.6)	63 (52.1)	26 (56.5)
KOOS Sport/Rec (mean, SD)			
0–100	39.3 (31.5)	42.9 (31.1)	30.1 (31.1)
≤85.0 (*n*, %)	144 (89.4)	101 (83.5)	43 (93.5)
KOOS QoL (mean, SD)			
0–100	59.6 (26.6)	63.3 (24.4)	49.9 (29.9)
≤87.5 (*n*, %)	138 (84.1)	98 (81.0)	40 (87.0)

After TKA surgery, 46 patients (38 %) never returned to work (Table 1). Eight (17 %) reported that this was due to their TKA, seven (15 %) reported other physical complaints, and twenty-six reported that they had “retired” (57 %). One hundred and twenty-one (72 %) patients returned to work, of which eight (7 %) within 1 month, 50 (41 %) between 1 and 3 months, 43 (36 %) within 3–6 months, and 20 (17 %) after 6 months. Of these TKA patients, 19 reported that they had a less physically demanding job after RTW, 79 had an equally physically demanding job, and 12 had a more physically demanding job after RTW. Regarding working hours, 11 patients reported fewer working hours, 96 reported the same number of working hours, and five reported more working hours.

### Factors associated with no RTW

Multicollinearity was not present for the included factors. Five distinct patient characteristics remained in the final model for no RTW (Table 2). The strongest association with no RTW was found for preoperative sick-leave duration of more than two weeks (OR 12.5, 90 % CI 5.0–31.5). Patient-reported work-relatedness of the knee symptoms finally resulting in TKA had the second highest OR of 5.3 (90 % CI 2.0–14.1). The other three were: a medium physically knee-demanding job (OR 3.3, 90 % CI 1.2–8.9), female sex (OR 3.2, 90 % CI 1.3–8.2), and BMI ≥ 30 (OR 2.8, 90 % CI 1.1–7.1). This model explained 50 % of the variance (Nagelkerke *R*^2^ = 0.50).

**Table 2:** The five predictors remaining in the final model after backward stepwise logistic regression for not returning to work (no RTW) including odds ratios (OR) and 90 % confidence intervals (CI)

Predictors for no RTW	Reference	OR	90 % CI	
Preoperative sick-leave duration >2 weeks	0–2 weeks	12.5	5.0	31.5
Work-relatedness of knee symptoms (Yes)	No	5.3	2.0	14.1
Medium knee-demanding job	Light	3.3	1.2	8.9
Female	Male	3.2	1.3	8.2
Body mass index ≥30.0	<30	2.8	1.1	7.1

## Discussion

TKA is being performed on an increasingly younger population of knee OA patients for whom participating in work is of critical importance. This study showed that KOOS pain, symptoms, ADL, and Sport/Rec were not associated with no RTW after TKA. Therefore, clinicians should be aware that proxies for participating in work go beyond outcomes like pain or function [6]. Additionally, the standardized care pathways after TKA focussing on minimizing pain and maximizing function like improving strength and mobility are probably not suited to overcoming hindering factors for RTW. A focus on rehabilitation on the performance of relevant work-related knee-demanding activities might be more promising, given the reported limitations in these activities before and after TKA [5].

Five predictors for no RTW among TKA patients were found in the present study. The strongest was having had a preoperative sick leave >2 weeks. This highlights the need for a better understanding of why these patients were on sick leave, and whether earlier TKA surgery in these patients might improve RTW due to a better preoperative health status and functioning [8, 19]. In line with the two former multivariate studies, this study showed that physical job demands hinder RTW [8, 9]. Interestingly, this association was established only for medium knee-demanding work and not for heavy knee-demanding work. This appears in line with Lombardi et al., who found that the highest percentage of patients that were still working at 1 year after TKA were those in very heavily demanding jobs: 98 %, 135 out of 138 patients [6]. An explanation might be the healthy worker selection effect. This means that, despite their TKA, this selected group of workers is more fit than the selection of workers involved in medium knee-demanding work. The reason is that unfit workers would have left their heavy knee-demanding work in an earlier phase in their career due to health complaints than their counterparts in medium knee-demanding work. This study also confirmed that sex is not associated with no RTW for males [9], but the opposite is true for females [8]. We can only speculate on the actual underlying reasons for this association; perhaps the fact that most men are the primary wage earners or that women in general have poorer outcomes after TKA due, for instance, to depression, low back pain, and symptomatic joint count [20]. A BMI ≥ 30 and having a TKA might further reduce sports participation and thereby increase the risk for no RTW [21, 22]. The fifth predictor for no RTW was the self-reported work-relatedness of symptoms leading to the TKA. This patient characteristic has not been reported in other joint replacement studies on RTW. Interestingly, this characteristic was not associated with the classification of the job into light-, medium-, and heavy knee-demanding work, and perhaps it is associated with the motivation of TKA patients for RTW [9]. Taken together, these five predictors explained 50 % of no RTW: a relatively high impact. To improve the ease of use of these predictors in a clinical setting, the corresponding patient characteristics were dichotomized or trichotomized. These predictors can guide clinicians to select patients at risk for no RTW. For instance, a plausible first step seems to be active referral of target patients characterized by the above-mentioned predictors to an occupational physician. Preferably, this should be done preoperatively TKA to secure timely work-directed care.

Two limitations of the present study should be discussed. The first limitation is the potential presence of recall bias. To reduce this bias, we categorized the answers, most often in two categories and not more than three. In addition, to overcome differences due to follow-up time between TKA surgery and filling in the questionnaire between patients, this period was included in the regression analysis and appeared not to be associated with no RTW. A second limitation is the relatively small number of patients that did not RTW, resulting in less precision of the risk estimates: 46 patients (28 % of 167) in the present study with a follow-up of at least 2 years. However, in the previous multivariate studies on RTW after TKA, the absolute number of patients not returning to work was even less: 45 (28 % of 162) TKA patients not returning to work at the 3-month postoperative end point [9] and 26 (15 % of 170) TKA patients not returning to work at the 12-month postoperative end point [8]. Given the estimated increasing number of working TKA patients in the coming years, multivariate prognostic studies on RTW with sufficient power are needed to critically understand the disease-specific mechanisms for no RTW, including relevant comorbidity [23]. Meanwhile, patients at risk for no RTW—especially obese female workers with a preoperative sick-leave duration >2 weeks who perform knee-demanding work and indicate that their knee symptoms are work-related, should actively be referred for work-directed care.

## References

[1] Skou ST, Roos EM, Laursen MB (2015). A randomized, controlled trial of total knee replacement. N Engl J Med.

[2] Kurtz SM, Lau E, Ong K, Zhao K, Kelly M, Bozic KJ (2009). Future young patient demand for primary and revision joint replacement: national projections from 2010 to 2030. Clin Orthop Relat Res.

[3] Otten R, van Roermund PM, Picavet HSJ (2010). Trends in the number of knee and hip arthroplasties: considerably more knee and hip prostheses due to osteoarthritis in 2030. Ned Tijdschr Geneeskd.

[4] Culliford D, Maskell J, Judge A, Cooper C, Prieto-Alhambra D, Arden NK, COASt Study Group (2015). Future projections of total hip and knee arthroplasty in the UK: results from the UK clinical practice research datalink. Osteoarthr Cartil.

[5] Kievit AJ, van Geenen RC, Kuijer PP, Pahlplatz TM, Blankevoort L, Schafroth MU (2014). Total knee arthroplasty and the unforeseen impact on return to work: a cross-sectional multicenter survey. J Arthroplasty.

[6] Lombardi AV, Nunley RM, Berend KR (2014). Do patients return to work after total knee arthroplasty?. Clin Orthop Relat Res.

[7] Abasolo L, Lajas C, León L (2012). Prognostic factors for long-term work disability due to musculoskeletal disorders. Rheumatol Int.

[8] Sankar A, Davis AM, Palaganas MP, Beaton DE, Badley EM, Gignac MA (2013). Return to work and workplace activity limitations following total hip or knee replacement. Osteoarthr Cartil.

[9] Styron JF, Barsoum WK, Smyth KA, Singer ME (2011). Preoperative predictors of returning to work following primary total knee arthroplasty. J Bone Joint Surg Am.

[10] Tilbury C, Leichtenberg CS, Tordoir RL (2015). Return to work after total hip and knee arthroplasty: results from a clinical study. Rheumatol Int.

[11] Malviya A, Wilson G, Kleim B, Kurtz SM, Deehan D (2014). Factors influencing return to work after hip and knee replacement. Occup Med (Lond).

[12] Palmer KT (2012). The older worker with osteoarthritis of the knee. Br Med Bull.

[13] Kuijer PP, de Beer MJ, Houdijk JH, Frings-Dresen MH (2009). Beneficial and limiting factors affecting return to work after total knee and hip arthroplasty: a systematic review. J Occup Rehabil.

[14] Kievit AJ, Schafroth MU, Blankevoort L, Sierevelt IN, van Dijk CN, van Geenen RC (2014). Early experience with the Vanguard complete total knee system: 2–7 years of follow-up and risk factors for revision. J Arthroplasty.

[15] Tilbury C, Schaasberg W, Plevier JW, Fiocco M, Nelissen RG, Vliet Vlieland TP (2014). Return to work after total hip and knee arthroplasty: a systematic review. Rheumatology (Oxford).

[16] Kleim BD, Malviya A, Rushton S, Bardgett M, Deehan DJ (2015). Understanding the patient-reported factors determining time taken to return to work after hip and knee arthroplasty. Knee Surg Sports Traumatol Arthrosc.

[17] Englund M, Roos EM, Lohmander LS (2003). Impact of type of meniscal tear on radiographic and symptomatic knee osteoarthritis: a sixteen-year follow up of meniscectomy with matched controls. Arthritis Rheum.

[18] McWilliams DF, Leeb BF, Muthuri SG, Doherty M, Zhang W (2011). Occupational risk factors for osteoarthritis of the knee: a meta-analysis. Osteoarthr Cartil.

[19] Kievit AJ, Kuijer PP, Kievit RA, Sierevelt IN, Blankevoort L, Frings-Dresen MH (2014). A Reliable, Valid and Responsive Questionnaire to score the impact of knee complaints on work following total knee arthroplasty: the WORQ. J Arthroplasty.

[20] Mehta SP, Perruccio AV, Palaganas M, Davis AM (2015). Do women have poorer outcomes following total knee replacement?. Osteoarthr Cartil.

[21] Robroek SJ, Rongen A, Arts CH, Otten FW, Burdorf A, Schuring M (2015). Educational inequalities in exit from paid employment among Dutch workers: the influence of health, lifestyle and work. PLoS One.

[22] Witjes S, Gouttebarge V, Kuijer PP, van Geenen RC, Poolman RW, Kerkhoffs GM (2016). Return to sports and physical activity after total and unicondylar knee arthroplasty: a systematic review and meta-analysis. Sports Med.

[23] Peter WF, Dekker J, Tilbury C (2015). The association between comorbidities and pain, physical function and quality of life following hip and knee arthroplasty. Rheumatol Int.

